# Astragalus polysaccharides inhibit ovarian cancer cell growth via microRNA-27a/FBXW7 signaling pathway

**DOI:** 10.1042/BSR20193396

**Published:** 2020-03-27

**Authors:** Yanling Guo, Zhenxing Zhang, Zhaoxia Wang, Guoqi Liu, Yingying Liu, Huijie Wang

**Affiliations:** 1 Oncology Department of Integrative Medicine, Luoyang Central Hospital Affiliated to Zhengzhou University, Luoyang 471009, Henan, China; 2 Department of Nutriology, Luoyang Central Hospital Affiliated to Zhengzhou University, Luoyang 471009, Henan, China

**Keywords:** Astragalus polysaccharides, apoptosis, FBXW7, microRNA-27a, ovarian cancer

## Abstract

Astragalus polysaccharide (APS), a natural antioxidant found in Astragalus membranaceus emerging as a novel anticancer agent, exerts antiproliferative and pro-apoptotic activity in various cancer cell types, but its effect on ovarian cancer (OC) remains unknown. In the present study, we tried to elucidate the role and mechanism of APS in OC cells. Our results showed that APS treatment suppressed the proliferation and induced apoptosis in OC cells. Afterward, the microRNA (miRNA) profiles in APS-treated cells were determined by a microarray assay, and whether APS affected OV-90 cells through regulation of miRNA was determined. Among these aberrant miRNAs, miR-27a was selected for further study as its oncogenic roles in various human cancers. Moreover, we found overexpression of miR-27a reversed the antiproliferation and pro-apoptotic effects of APS on OC cells. F-box and WD-40 domain protein 7 (FBXW7), a classical tumor suppressor, was found directly targeted by miR-27a and its translation was suppressed by miR-27a in OC cells. Finally, it was also observed that knockdown of FBXW7 by si-FBXW7 reversed the tumor suppressive activity of APS in OC cells, which is similar to the effects of miR-27a overexpression. Our findings demonstrate that APS can suppress OC cell growth *in vitro* via miR-27a/FBXW7 axis, and this observation reveals the therapeutic potential of APS for treatment of OC.

## Introduction

Ovarian cancer (OC) is one of the most common gynecological malignancies in women and the incidence rate is right next to cervical cancer and endometrial cancer [1,2]. Although great improvements have been made, the 5-year survival rate of this disease is only 30%, due to the recurrence and severe metastasis [3,4]. Therefore, the need for more effective therapeutic drug has become a priority.

Astragalus polysaccharides (APS), an active ingredient isolated from the roots of Astragalus membranaceus, has gained considerable attention due to its potent anticancer activities. APS has been shown to be remarkable in inhibiting cell proliferation and inducing apoptosis of human breast cancer and osteosarcoma [5,6]. For example, APS treatment suppressed the tumor growth in mice model of lung cancer [7]. APS inhibited the growth of nasopharyngeal carcinoma (NPC) cells and enhanced the therapeutic effect when combined with cisplatin in the xenograft model [8]. A clinical study has indicated the beneficial roles of APS combined with chemotherapy in advanced non-small-cell lung cancer (NSCLC) patients [9], with few associated side effects; however, the role and mechanisms of APS in OC remains unclear.

MicroRNAs (miRNAs) are single-stranded, non-coding RNAs that bind to the target mRNAs and interfere in the translation process [10]. A large number of miRNAs have been reported to be involved in the progression of OC, acting as oncogenes or tumor suppressors. For example, Xie et al. [11] found that miR-663 promoted the cell viability and invasion in OC cells through inhibiting TUSC2. Hua et al. [12] showed that miR-145 overexpression exhibited a suppressive effect on the OC cell proliferation, migration and invasion. Notably, a previous study has shown that in human osteosarcoma (OS), APS treatment decreased the OC cell proliferation and induced their apoptosis by up-regulation of a tumor suppressive miRNA, miR-133a [6]. Thus, we hypothesized that APS might affect OC cells through regulation of miRNAs.

In the present study, we examined the effects of APS on the proliferation and apoptosis of OC cells. Moreover, the underlying molecular mechanism involved in the anti-tumor actions of APS was investigated. Our findings provide a theoretical basis for further exploration of APS in the treatment of OC.

## Materials and methods

### Cell culture and treatment

Human OC cell lines OV-90 and SKOV-3, and HEK 293T cells were obtained from ATCC (Manassas, VA, U.S.A.). All cells were maintained in DMEM (Invitrogen, U.S.A.) containing 10% FBS (Gibco, Grand Island, NY, U.S.A.) in a 5% CO_2_ incubator at 37°C.

APS were purchased from Sigma–Aldrich (St. Louis, MO, U.S.A.). APS dissolved in DMEM ranging from 0 to 2 mg/ml were added into OV-90 and SKOV-3 cells at 37°C for 24 h.

### Cell proliferation

The antiproliferative effect of APS against OC cells was determined using MTT assay. At the end of treatment, 20 μl MTT solution (Sigma–Aldrich) was added to each well (1 × 10^5^/well), and OV-90 and SKOV-3 cells were cultured for another 2 h. Then the OD absorbance of the samples at 450 nm was detected by iMark microplate reader (Bio-Rad, Hercules, CA, U.S.A.).

### Cell apoptosis

The apoptosis portion was assessed by Annexin V-FITC Apoptosis Detection Kit (Beyotime, China). At the end of treatment, OV-90 and SKOV-3 cells were harvested and re-suspended in binding buffer at room temperature (RT) in the dark for 15 min. The samples were done by a FACSCalibur flow cytometer (BD Biosciences) and then analyzed by FlowJo software (Version 8.7.1, Tree Star, Ashland, OR, U.S.A.).

### Transwell invasion assay

Transwell chambers (8-μm pore; BD Biosciences) coated with Matrigel (BD Biosciences, San Jose, CA) were utilized for invasion assay. Briefly, OV-90 and SKOV-3 (8 × 10^4^) were seeded in the top chamber with serum-free medium, while the lower chamber was added with the culture medium with 20% FBS. After 24-h incubation, the cells were fixed and incubated in 1% Crystal Violet solution for 10 min for staining. An inverted microscope (IX71, Olympus Corp., Tokyo, Japan) was used to observe and photograph the cells that had migrated into the lower chamber of the Transwell system at ×200 magnification.

### Wound healing assay

When OV-90 and SKOV-3 cells reached approximately 80% confluence, the monolayer was scratched with a 10-μl pipette tip at the time recorded as 0 h. After incubation at 37°C for 24 h, the culture medium was removed, and the plate was washed with PBS buffer three times. The cell debris produced during scratching was washed away, followed by the addition of serum-free culture medium and imaging at 24 h. Then the ImageJ analysis software (Bethesda, MD, U.S.A.) was used to calculate the migration distances.

### The caspase-3 activity

After various treatments for 24 h, caspase-3 activity in OV-90 and SKOV-3 cells was measured using a caspase-3 activity assay kit (Beyotime, Shanghai, China).

### Immunofluorescence

After APS treatment, the cells were fixed in absolute ethyl alcohol for 30 min at RT. After washing twice with PBS, the fixed cells were stained with primary antibody Bax (1:100; cat no. ab32503; Abcam, Cambridge, U.K.) for 1 h at RT. Then, the secondary antibody conjugated with FITC (1:200, cat no. ab116639; Abcam, Cambridge, U.K.) was added for 2 h in the dark, fluorescence images were collected and analyzed using an inverted fluorescence microscope.

### miRNA microarray

Total RNA was extracted from APS treated and untreated OV-90 cells by miRNeasy isolation kit (Qiagen, Milan, Italy) according to the manufacturer’s protocol and the miRNA fraction was further purified by a mirVana miRNA isolation kit (Ambion, Austin, TX). miRCURY LNA™ microRNA Array with 1223 probes containing 3000 capture probes representing all human, mouse and rat microRNAs’ sequences were used to screen differentially expressed miRNAs between APS treated and untreated OV-90 cells. Total RNA (200 ng) was labeled using the miRCURY LNA™ Hy3™/Hy5™ Power labeling kit (Exiqon, Vedbaek, Denmark) and hybridized on the miRCURY™ LNA Array (v.16.0) (Exiqon) according to the manufacturer’s protocol. Microarray images were taken with a Genepix 4000B scanner (Axon Instruments, Foster City, CA, U.S.A.) and analyzed with Genepix Pro 6.0 software (Axon Instruments). Finally, the heat map of the 52 microRNAs most obvious differences was created using a method of hierarchical clustering by GeneSpring GX, version 7.3 (Agilent Technologies, California, United States). The threshold of screening differentially expressed miRNAs was set as a fold change >2.0 and a *P*-value <0.05. The microarray data that support the findings of the present study are available from the corresponding author upon reasonable request.

### qRT-PCR

Total RNA was extracted from cells with the TRIzol reagent (Invitrogen, U.S.A.). Reverse transcription of miR-27a and F-box and WD-40 domain protein 7 (FBXW7) was synthesized using the miScript II RT kit and the reverse transcription kit (Invitrogen, Carlsbad, CA), respectively. miR-27a and FBXW7 expressions were measured using the Exiqon SYBR Green Master Mix (Exiqon, Vedbaek, Denmark) on a Light Cycler instrument (Bio-Rad). The primers used were as follows: miR-27a F: 5′-CCCAAGCTTACTGTGAAACTGTGAAACGTGAAACTGTGAAACTGTGAAACTGTGAATCTAGAGC-3′; miR-27a R: 5′-GCTCTAGATTTCA-3′; U6 F: 5′-TGCGGGTGCTCGCTTCGCAGC-3′; U6 R: 5′-CCAGTGCAGGGTCCGAGGT-3′; FBXW7 F: 5′- GTCCCGAGAAGCGGTTTGATA-3′, FBXW7 R: 5′-TGCTCAGGCACGTCAGAAAAG-3′; GAPDH F: 5′-AGGTCGGTGTGAACGGATTTG-3′, GAPDH R: 5′-TGTAGACCATGTAGTTGAGGTCA-3′. Relative quantification was determined by normalization to U6 or GAPDH. The qRT-PCR assays were performed in triplicate and the relative expression levels were calculated based on the 2^−ΔΔ*C*_t_^ method [13].

### Cell transfection

MiR-27a mim[ics, miR-27a inhibitor, the corresponding control vectors, si-FBXW7 and si-scramble were obtained from RiboBio Co., Ltd. (Guangzhou, China). Cell transfection was performed using Lipofectamine® 2000 (Invitrogen) when OV-90 and SKOV-3 in six-well plate grown to approximately 80% confluence following the manufacturer’s protocol. Twenty-four hours post transfection, the cells were stimulated with APS for 24 h and then utilized in subsequent experiments. The sequences of miR-27a mimics/inhibitor are as follows: miR-27a mimics: 5′-UUCACAGUGGCUAAGUUCCGC-3′; miR-27a inhibitor: 5′-GCGGAACUUAGCCACUGUGAA-3′, mimics NC: 5′-UUCUCCGAACGUGUCACGUTT-3′, inhibitor NC: 5′-CAGUACUUUUGUGUAGUACAA-3′.

### Luciferase reporter assay

pGL3-FBXW7 wild-type (wt) or pGL3-FBXW7 mutant type (mut) were co-transfected with miR-27a mimics into HEK 293T cells in 24-well plates (2 × 10^5^/well) using Lipofectamine 2000 (Invitrogen). At 24 h post-transfection, the double luciferase activities were analyzed using the Dual-Luciferase Reporter Assay system (Promega Corporation).

### Western blot

Western blotting was performed as previously described [14]. Briefly, 40 μg extracted protein samples were separated by 12% SDS/PAGE (w/v) and transferred on to a PVDF membrane (Millipore). Then, the membranes were blocked with 5% skim milk for 2 h at RT, followed by incubation with primary antibodies against Bax (1:1000; cat no. ab32503; Abcam, Cambridge, U.K.), FBXW7 (cat no. ab227677; 1:1000, Abcam, Cambridge, U.K.), E-cadherin (cat no. #3195; 1:1000, Cell Signaling, Danvers, MA, U.S.A.), N-cadherin (cat no. #13116; 1:1000, Cell Signaling, Danvers, MA, U.S.A.), Fibronectin (cat no. #26836; 1:1000, Cell Signaling, Danvers, MA, U.S.A.), Vimentin (cat no. #5741; 1:1000, Cell Signaling, Danvers, MA, U.S.A.) and β-actin antibody (cat no. #4970; 1:1000, Cell Signaling, Danvers, MA, U.S.A.) at 4°C overnight, followed by HRP-conjugated goat anti-rabbit IgG (cat. no. 205718; 1:10,000, Abcam, Cambridge, U.K.). The protein bands were developed using ECL kit (GE Healthcare) and blot bands were quantified with ImageJ version 1.46 (Rawak Software, Inc. Munich, Germany).

### Statistical analysis

Statistical analysis was performed using GraphPad Prism (version 5.0, GraphPad Software, Inc., La Jolla, CA, U.S.A.). Each experiment was repeated at least three times. Data were recorded as means ± SD. One-way analysis of variance (ANOVA) with a Bonferroni correction were used to analyze differences between groups. A *P*-value <0.05 was considered significant.

## Results

### APS inhibited cell proliferation and induced cell apoptosis in OC cells

**Figure 1:**
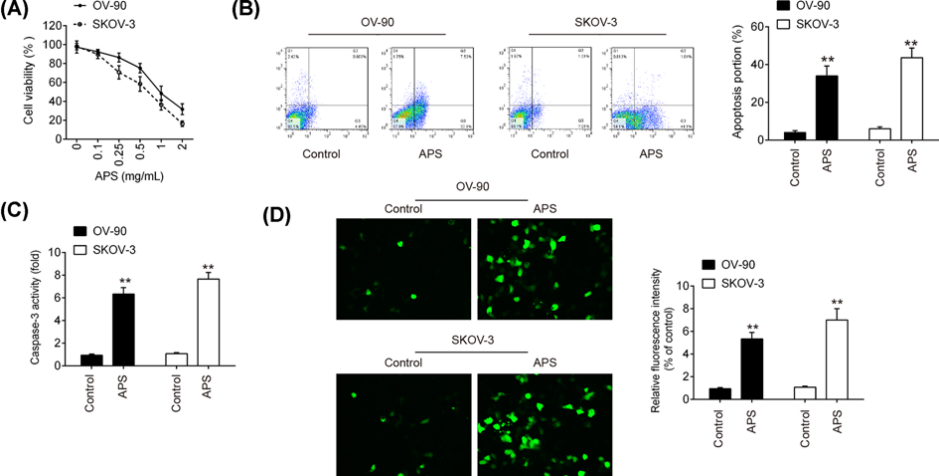
APS reduced proliferation and induced apoptosis of OV-90 and SKOV-3 cells OV-90 and SKOV-3 cells were treated with 0–2 mg/ml APS for 24 h. Non-treated cells served as control. (**A**) Cell viability was measured by MTT assay. (**B**) The apoptosis was detected by flow cytometry. (**C**) The activity of caspase-3 was determined by a commercial kit. (**D**) Expression level of apoptosis-associated protein Bax was evaluated by immunofluorescence. Data were represented as the mean ± SD of three independent experiments. **P<0.01 vs. Control group.

Initially, to investigate the effects of APS on cell proliferation, OV-90 and SKOV-3 cells were treated with different concentrations of APS (0–2 mg/ml). As shown in Figure 1A, cell viability was significantly reduced after APS treatment, compared with control group, and this inhibitory effect was dose-dependent and 1 mg/ml APS exhibited the best promoting effect. Therefore, the APS concentration of 1 mg/ml was chosen for subsequent experiments. Then, the effect of APS on the cell apoptosis was measured using flow cytometry assay. The results showed that APS markedly increased the apoptotic portions of OV-90 and SKOV-3 cells, compared with control group (Figure 1B). Meanwhile, the caspase-3 activity and the expressions of Bax were significantly increased after APS treatment, as determined by a caspase-3 activity assay and immunofluorescence (Figure 1C,D). Collectively, these results suggest that APS may inhibit cell viability through inducing cell apoptosis in OC cells.

### APS inhibited cell invasion and migration in OC cells

**Figure 2:**
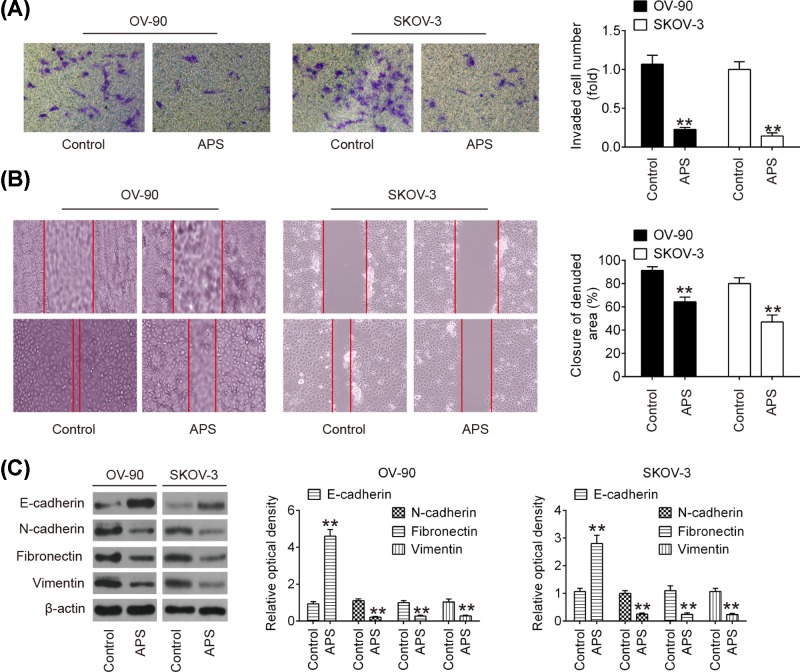
APS inhibited cell invasion and migration in OC cells (**A**) The invasion of OV-90 and SKOV-3 cells after APS treatment was measured by Transwell assay. (**B**) The migration of OV-90 and SKOV-3 cells after APS treatment was measured by Wound healing assay. (**C**) The expression of EMT proteins in OV-90 and SKOV-3 cells after APS treatment was detected by Western blot. Data were represented as the mean ± SD of three independent experiments. **P<0.01 vs. Control group.

Next, to determine whether APS also affects the invasive and migratory abilities of OC cells, transwell and wound healing assays were performed. As shown in Figure 2A, compared with the control group, APS treatment significantly suppressed the invasive ability of both OV-90 and SKOV-3 cells. Moreover, wound healing assay showed that APS treatment markedly inhibited wound closures compared with the control group (Figure 2B). To further examine the role of APS in cell migration and invasion in OC cells, epithelial–mesenchymal transition (EMT)-related proteins were analyzed by Western blot. As shown in Figure 2C, APS treatment significantly increased E-cadherin expression and inhibited N-cadherin, Fibronectin and vimentin expressions compared with control group. Collectively, these data indicated that APS inhibited cell invasion and migration by regulating expression of EMT-related proteins in OC cells.

### APS down-regulated the expression of miR-27a in OC cells

**Figure 3:**
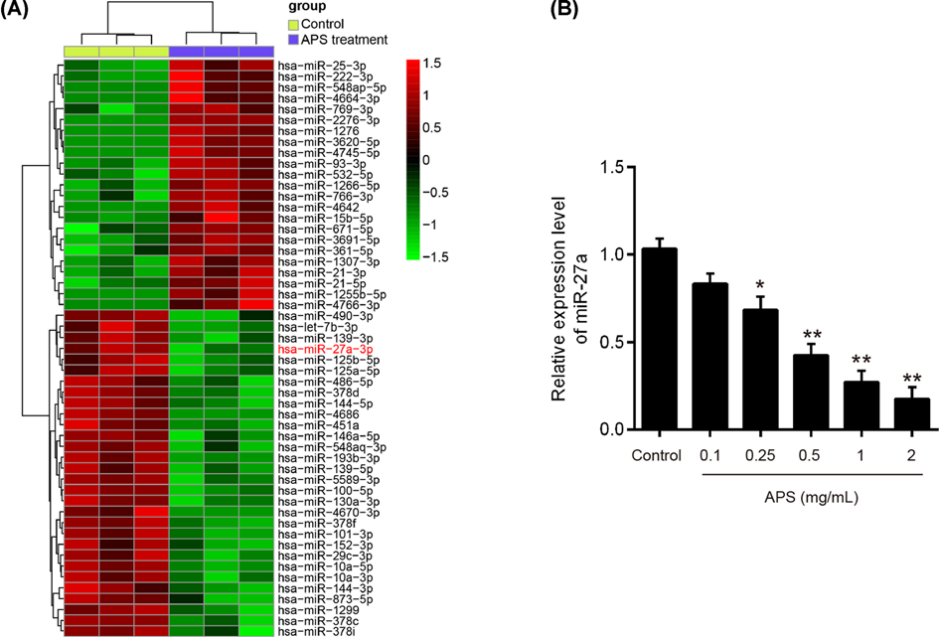
APS induced down-regulation of miR-27a in OV-90 cells OV-90 cells were treated by 1 mg/ml APS for 24 h. Non-treated cells served as control. (**A**) Heat map of miRNA profiles represents the differentially expressed miRNAs between APS-treated cells and control cells. Green indicates low expression levels; red indicates high expression levels. (**B**) The expression of miR-27a was detected by qRT-PCR after different concentrations APS (0–2 mg/ml) treatment. Data were represented as the mean ± SD of three independent experiments. *P<0.05, **P<0.01 vs. Control group.

Several studies have reported that APS exhibits its growth inhibitory effects on cancer cells through modulation of miRNAs [6,15]. Based on the studies reported above, we postulated that APS induced antiproliferative and pro-apoptotic effects in OC through regulation of miRNAs. An miRNA microarray was performed to screen the miRNAs that were regulated by APS. As shown in Figure 3A, 23 miRNAs were up-regulated and 29 miRNAs were down-regulated after APS treatment in OV-90 cells. Among them, miR-27a was selected for further investigation as it was the most significantly up-regulated miRNA in the APS treatment group. Interestingly, miR-27a has been identified as oncogenic miRNA in OC, with marked effects on cell growth of OC cells [16–18]. Thus, we sought to determine the potential roles of miR-27a in the actions of APS in subsequent studies.

To confirm the results from microarray, we detected the expressions of miR-27a in APS (0–2 mg/ml) treated OV-90 cells by qRT-PCR. The result showed that APS dose-dependently decreased the levels of miR-27a in OV-90 cells (Figure 3B). All data indicate that miR-27a is a potential target of APS in OC cells.

### Overexpression of miR-27a reversed the anti-tumor effect of APS in OC cells

**Figure 4:**
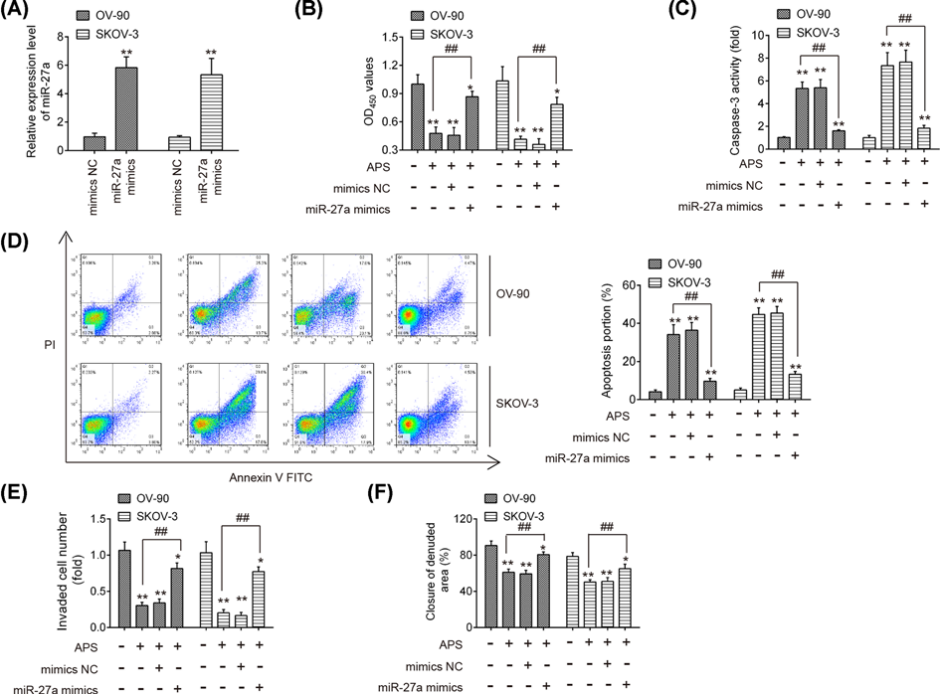
Overexpression of miR-27a reversed the anti-tumor effect of APS in OC cells miR-27a mimics was transfected into OV-90 and SKOV-3 cells 24 h prior to APS treatment. (**A**) The transfection efficiency of miR-27a was determined by qRT-PCT. (**B**) Cell viability was measured by MTT assay. (**C**) The activity of caspase-3 was determined by a commercial kit. (**D**) The apoptosis was detected by flow cytometry. (**E**) The invasion of OV-90 and SKOV-3 cells was measured by Transwell assay. (**F**) The migration of OV-90 and SKOV-3 cells was measured by Wound healing assay. Data were represented as the mean ± SD of three independent experiments. *P<0.05, **P<0.01 vs. Control group, ^##^*P*<0.01 vs. APS group.

To further examine whether APS exerts its antiproliferative and pro-apoptotic effects through regulating miR-27a expression, miR-27a mimics were transfected into OV-90 and SKOV-3 cells 24 h prior to APS treatment. The qRT-PCR analysis showed that the expression of miR-27a was notably increased after miR-27a mimics transfection (Figure 4A). Using MTT assay, it was observed that the reduction in cell viability caused by APS treatment was restored by overexpression of miR-27a (Figure 4B). Meanwhile, miR-27a overexpression reversed the promoting effects of APS on caspase 3 activity and cell apoptosis in OV-90 and SKOV-3 cells (Figure 4C,D). Additionally, the inhibitory effects of APS on the invasion and migration of OV-90 and SKOV-3 cells were also significantly reversed by miR-27a overexpression (Figure 4E,F). These data suggest that miR-27a is involved in the anti-cancer effects of APS on OC cells.

### FBXW7 was a direct target of miR-27a

**Figure 5:**
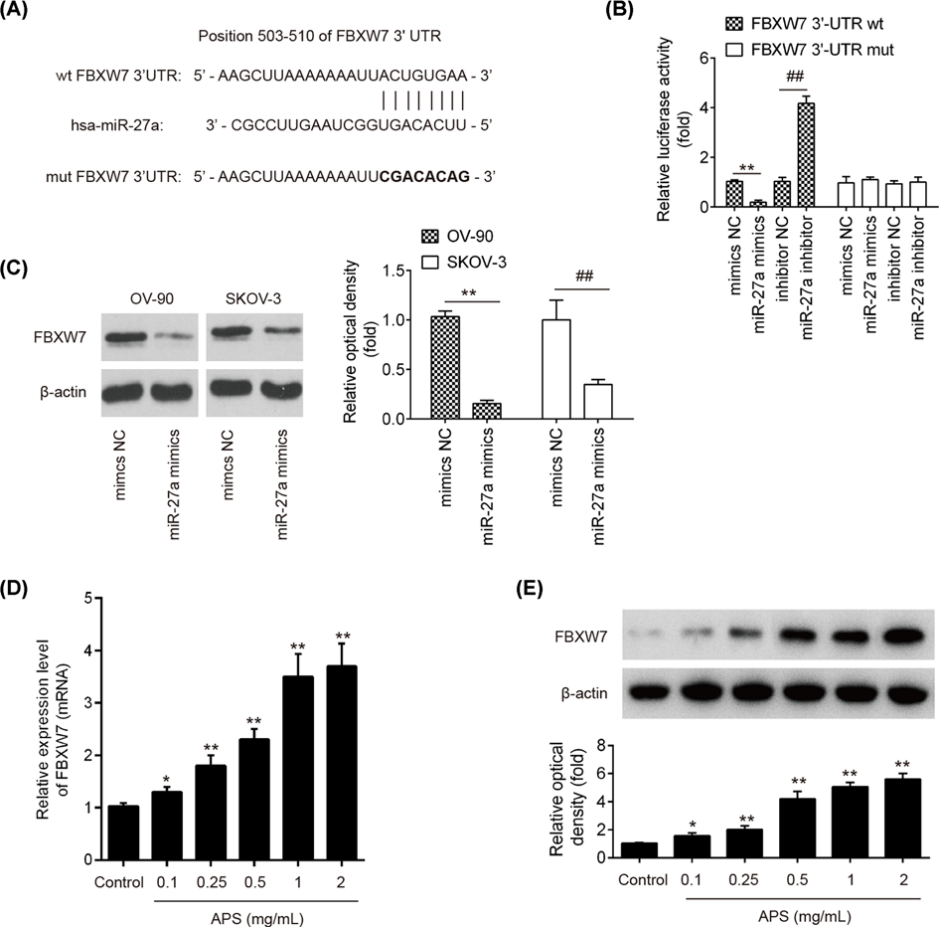
FBXW7 was a direct target of miR-27a (**A**) The putative binding site of miR-27a and FBXW7 is shown. (**B**) Luciferase assay of OV-90 cells co-transfected with firefly luciferase constructs containing the FBXW7 wild-type or mutated 3′-UTRs and miR-27a mimics, mimics NC, miR-27a inhibitor or inhibitor NC, as indicated (*n*=3). Data represent the mean ± SD of three independent experiments. **P<0.01 vs. mimics NC; ^##^*P*<0.01 vs. inhibitor NC. (**C**) OV-90 and SKOV-3 were transfected with miR-27a mimics or negative control for 24 h, then the cells collected and the protein level of FBXW7 was analyzed by Western blot. (**D,E**) The mRNA and protein expressions of FBXW7 were measured by qRT-PCR and Western blot after different concentrations APS (0–2 mg/ml) treatment. Data represent the mean ± SD of three independent experiments. *P<0.05, **P<0.01 vs Control group; ^##^*P*<0.01 vs APS group.

To explore the underlying molecular mechanisms involved in the miR-27a-mediated anti-tumor activity of APS in OC cells, we relied on TargetScan 7.0 and miRanda to predict the targets of miR-27a and identified FBXW7 as a potential target of miR-27a (Figure 5A), which has previously been reported to be a tumor suppressor in various types of human cancers. To confirm the relationship between FBXW7 and miR-27a, a luciferase reporter assay was performed. The results revealed that transfection with miR-27a mimics significantly attenuated the luciferase activity of the FBXW7-3′UTR wt reporter plasmid, whereas the miR-27a inhibitor transfection increased its luciferase activity; however, no effects on the mutated version of FBXW7 3′UTR was observed (Figure 5B). Furthermore, miR-27a overexpression decreased protein level of FBXW7 in OV-90 and SKOV-3 cells, as determined by Western blot analysis (Figure 5C), suggesting that miR-27a suppressed the translation of FBXW7 in OC cells. In addition, it was demonstrated that APS dose-dependently increased the expression of FBXW7 in OV-90 cells at mRNA and protein levels (Figure 5D,E). All data suggest that APS may exert its anti-tumor action through liberating the suppressive effect of miR-27a on FBXW7.

### Knockdown of FBXW7 reversed the anti-tumor effects of APS in OC cells

**Figure 6:**
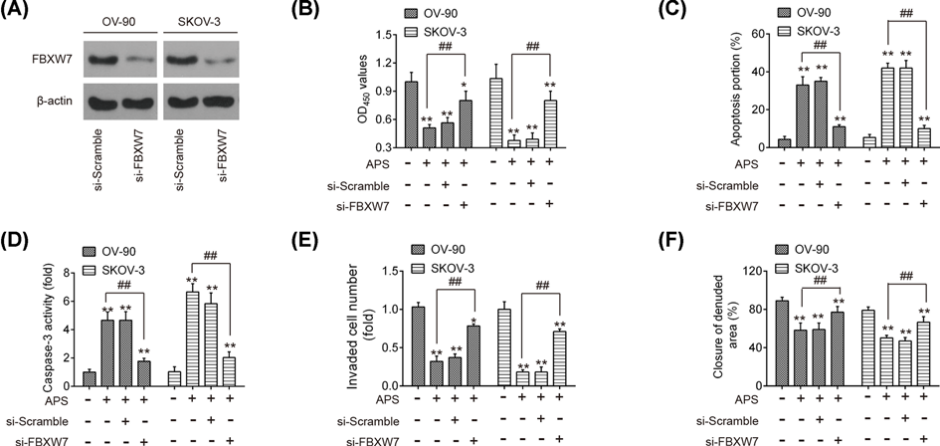
Knockdown of FBXW7 reversed the anti-tumor effects of APS in OC cells Si-FBXW7 was transfected into OV-90 and SKOV-3 cells 24 h prior to APS treatment. (**A**) The transfection efficiency of FBXW7 was determined by Western blot. (**B**) Cell viability was measured by MTT assay. (**C**) The apoptosis was detected by flow cytometry. (**D**) The activity of caspase-3 was determined by a commercial kit. (**E**) The invasion of OV-90 and SKOV-3 cells was measured by Transwell assay. (**F**) The migration of OV-90 and SKOV-3 cells was measured by Wound healing assay. Data represent the mean ± SD of three independent experiments. *P<0.05, **P<0.01 vs Control group; ^##^*P*<0.01 vs APS group.

As mentioned above, APS was identified to dose-dependently increase FBXW7 expression, and it was hypothesized that APS exerts its anti-tumor effects by enhancing FBXW7 expression. Si-FBXW7 was transfected into OV-90 and SKOV-3 cells 24 h prior to APS treatment and the transfection efficiency was determined by Western blot. As shown in Figure 6A, FBXW7 was notably decreased after si-FBXW7 transfection. Furthermore, FBXW7 knockdown by si-FBXW7 reversed the antiproliferative action of APS in OV-90 and SKOV-3 cells (Figure 6B). It was also observed that knockdown of FBXW7 attenuated the promoting effects of APS on apoptosis and caspase-3 activity in these cells (Figure 6C,D). Meanwhile, the inhibitory effects of APS on the invasive and migratory abilities of OV-90 and SKOV-3 cells were also significantly reversed by knockdown of FBXW7 (Figure 6E,F). All data indicate that APS suppressed cell proliferation and induced cell apoptosis through FBXW7 *in vitro*.

## Discussion

In the present study, we observe that APS treatment can successfully suppress cell proliferation and promoted cell apoptosis in OC cells, thereby demonstrating that it is a highly potent anti-tumor agent. The expression of miR-27a was reduced by APS and it caused up-regulation of tumor suppressive gene, *FBXW7*, ultimately inhibited cell proliferation and induced apoptosis in OV-90 cells. Our study provided an insight into the anti-tumor mechanisms of APS and APS may serve as a promising agent for the treatment of OC.

Increasing evidence has demonstrated that APS participates in suppression of biological activities, such as proliferation, invasion and migration in various types of human cancers. For example, Zhou et al. have reported that APS inhibited cell proliferation and metastasis, and promoted apoptosis in NSCLC cell lines via the MAP4K3/mTOR signaling pathway [19]. The anti-tumor effects of APS have been reported in murine H22 hepatocarcinoma model [20]. APS treatment combined with vinorelbine and cisplatin significantly improved life quality of patients with advanced NSCLC [9]. Another study performed by Li et al. demonstrated APS synergistically promoted the anti-tumor effects of cisplatin on OC cells by activating the JNK pathway [21]. However, little reports have been paid on the role of APS alone in OC. In our study, we investigated the effects of APS on OC cells and found that APS also exerts its anti-tumor activity through its antiproliferative and pro-apoptotic effects. However, the possible molecular mechanism requires further investigation.

It has been reported that natural products exert their anti-tumor activities via modulation of various miRNAs. For example, curcumin, a type of polyphenol, has been found to inhibit cell growth through modulation of miR-99a in retinoblastoma cells [22]. Resveratrol could alter the expressions of many miRNAs in PC-3M-MM2 cells, which may contribute to its anti-tumor property in prostate cancer [23]. A recent study has demonstrated that APS play a tumor suppressive role in human osteosarcoma cells by up-regulation of miR-133a [6]. It is for that reason that we investigated the role of miRNA in the anti-tumor property of APS. Using a miRNA microarray assay, large numbers of miRNAs were found to be differently expressed in APS treated OC cells; in particular, miR-27a was the most down-regulating miRNA after APS treatment, and thus was selected for further study.

MiR-27a is a well-known oncogene in multiple tumor types, including OC [16,24]. For example, miR-27a was found to be overexpressed in bladder and renal cancer tissues, and promoted the cancer cells growth and metastasis [25,26]. Ding et al. showed that miR-27a antagomir injection inhibited the tumor growth of gastric cancer in a xenograft model [27]. In OC, miR-27a overexpression promoted the process of EMT through activating Wnt/β-catenin signaling pathway by targeting FOXO1 [17]. Thus, it was hypothesized that APS exhibits its anti-tumor effect through down-regulating miR-27a expression. In our study, miR-27a was dose-dependently decreased in response to APS treatment in OC cells. Moreover, we observed that miR-27 overexpression reversed the antiproliferative and pro-apoptotic effects of APS on OC cells. All these results suggest that miR-27a mediated the anti-tumor actions of APS in OC. Notably, besides miR-27a, many other miRNAs were also down-regulated after APS treatment, such as miR-490, let-7b and miR-139. Chen et al. have found that miR-490 inhibited OC tumorigenesis and progression by targeting CDK1 *in vitro* and *in vivo* [28]. Gao et al. have shown that let-7b functions as a tumor suppressor in OC [29]. Wang et al. have reported that miR-139-5p markedly suppressed the growth of tumors by repressing ROCK2 expression in nude mice [30]. Although the roles of these miRNAs in OC have previously been investigated, but whether these miRNAs are involved in the anti-tumor effects of APS still unknown. Therefore, more investigations are required to reveal the full mechanisms of the beneficial effects of APS in OC cells, which might not be limited to targeting miR-27a.

To further elucidate the potential mechanism by which miR-27a mediated the anti-tumor activity of APS against OC, bioinformatics analysis was performed to predicate the putative targets of miR-27a, and FBXW7 was predicted as a potential target of miR-27a. Of note, FBXW7 functions as a tumor suppressor in various types of human cancers, due to its capability to suppress cell growth, invasion and migration [31–33]. For example, FBXW7 overexpression can lead to the reduction in cell proliferation, migration and invasion in renal cancer [34], HCC [35], and gastric cancer [36]. In OC, FBXW7 was down-regulated in the OC tissues, and its low expression was negatively correlated with the malignant potential of OC [37]. Notably, a previous study showed that miR-27a promoted the growth of esophageal cancer by targeting FBXW7 [38]. Jiang et al. showed that miR-27a promoted cell migration and induced EMT by suppressing FBXW7 in breast cancer [39]. In our study, FBXW7 was validated as a target of miR-27a and its translation was suppressed by miR-27a in OC cells. It was also observed that APS treatment dose-dependently increased the levels of FBXW7 at mRNA and protein levels. In addition, the anti-tumor effects of APS on OC were also abrogated by the inhibition of FBXW7, suggesting that APS exhibits its anti-tumor actions through removing the suppressive effect of miR-27a on FBXW7.

In conclusion, the present study demonstrated that APS treatment reduced the expression of miR-27a, and in turn results in the up-regulation of the tumor suppressive gene, *FBXW7*, finally leading to the reduction in cellular proliferation and the induction of apoptosis in OC. Our results indicate that APS may offer potential therapeutic benefits for OC.

## Abbreviations

APS: Astragalus polysaccharide
EMT: epithelial–mesenchymal transition
FBXW7: F-box and WD-40 domain protein 7
FOXO: forkhead box transcription factors
GAPDH: Glyceraldehyde 3-phosphate dehydrogenase
HCC: hepatocellular carcinoma
HRP: horseradish peroxidase
MAP4K3: mitogen-activated protein kinase kinase kinase kinase 3
miRNA: microRNA
NSCLC: non-small-cell lung cancer
OC: ovarian cancer
OD: optical density
RT: room temperature
TUSC2: tumor suppressor candidate 2
